# Clinical characteristics and outcomes of COVID-19 patients in a tertiary hospital in Baguio City, Philippines

**DOI:** 10.5365/wpsar.2021.12.4.852

**Published:** 2021-11-11

**Authors:** Karen Joyce C. Cortez, Bernard A. Demot, Samantha S. Bartolo, Dexter D. Feliciano, Verna Moila P. Ciriaco, Imari Irish E. Labi, Denzelle Diane M. Viray, Jenna Charise M. Casuga, Karol Anne B. Camonayan-Flor, Precious Mae A. Gomez, Marie Ellaine N. Velasquez, Thea Pamela T. Cajulao, Jovy E. Nigos, Maria Lowella F. De Leon, Domingo P. Solimen, Angelita G. Go, Francis M. Pizarro, Larry C. Haya, Ray P. Aswat, Virginia B. Mangati, Caesar Noel I. Palaganas, Mylene N. Genuino, Kimberley M. Cutiyog-Ubando, Karen C. Tadeo, Marienelle L. Longid, Nowell Benedict C. Catbagan, Joel B. Bongotan, Beverly Anne T. Dominguez-Villar, Joeffrey B. Dalao

**Affiliations:** a Department of Internal Medicine, Baguio General Hospital and Medical Center, Baguio City, Philippines.

## Abstract

**Objective:**

Coronavirus disease 2019 (COVID-19), caused by severe acute respiratory syndrome coronavirus 2  (SARS-CoV-2), primarily targets the respiratory system. This study describes the characteristics associated with mortality among patients infected with SARS-CoV-2 at a single hospital in Baguio City, Philippines.

**Methods:**

We reviewed medical records (including history, laboratory results and treatment regimen) of 280 confirmed COVID-19 patients admitted to a single hospital during March–October 2020. Clinical characteristics and outcomes (frequency and type of complication, recovery rate and mortality) were evaluated. Multiple logistic regression was used to analyse factors associated with mortality.

**Results:**

The mean age of COVID-19 patients was 48.4 years and the female-to-male ratio was 1.8:1. Hypertension, cardiovascular disease (CVD) and diabetes were the most frequent comorbidities reported. Common presenting symptoms were respiratory and constitutional, with 41% of patients not reporting symptoms on admission. Patients with moderate, severe and critical disease comprised 45%, 8% and 4%, respectively. A total of 15% had complications, health care-associated pneumonia being the most frequent complication. The recovery rate was 95%; 5% of patients died, with multiorgan failure being the most common cause. The presence of CVD, chronic kidney disease, prolonged prothrombin time and elevated lactate dehydrogenase (LDH) were associated with mortality.

**Discussion:**

Most COVID-19 patients in our population had asymptomatic to moderate disease on admission. Mortality from COVID-19 was associated with having CVD, chronic kidney disease, elevated LDH and prolonged prothrombin time. Based on these results, we emphasize that people should take all necessary precautions to avoid infection with SARS-CoV-2.

Coronavirus disease 2019 (COVID-19), caused by severe acute respiratory syndrome coronavirus 2 (SARS-CoV-2), primarily targets the respiratory system. In December 2019, an epidemiological alert was released in China following a rise in cases of pneumonia of unknown cause. The Philippines announced its first confirmed case on 31 January 2020. (*1*, *2*) The World Health Organization (WHO) officially declared a global pandemic on 11 March 2020, by which time the Philippines already had 49 confirmed cases, largely in the National Capital Region. (*2*)

Baguio City is located north of Manila, within the Cordillera Central mountain range in northern Luzon. The estimated population is 345 000, with adults (aged 19–60 years) and those aged over 60 years comprising 52% and 6.6% of the population, respectively. (*3*) Leading causes of morbidity include hypertension, diabetes, bronchitis and asthma. (*4*)

The first confirmed case in Baguio City was recorded on the city’s ninth day of quarantine during March 2020, with local sustained transmission declared six months later. (*5*) Worldwide, by the end of October 2020, there were 43 623 111 confirmed cases and 1 161 311 deaths. At that time in the Philippines, cases had risen to 373 144 and deaths to 7053. Baguio City comprised 0.53% of confirmed cases and 0.37% of deaths nationwide. (*6*-*8*) COVID-19 patients in Baguio City were admitted and treated in six local hospitals and three community isolation units.

Many reports describing the characteristics and outcomes of COVID-19 in different settings are being published. In this study, we describe the clinical characteristics and outcomes of COVID-19 patients and the characteristics associated with mortality at one hospital in Baguio City, Philippines.

## Methods

We conducted a retrospective study of all patients aged over 18 years with COVID-19, confirmed by reverse transcription polymerase chain reaction (RT–PCR), who were admitted to a tertiary hospital that was one of the government-mandated COVID-19 referral hospitals in Baguio City, Philippines from 1 March to 27 October 2020.

A total of 371 patients were admitted during this period. Paediatric cases (*n* = 80) and cases dead on arrival (*n* = 9) were excluded. Charts were excluded if they lacked information on age, sex, travel history or exposure, official RT–PCR result, complete blood count or chest radiography (*n* = 2), leaving 280 charts for analysis. The following data were extracted: patient history, exposure, initial laboratory results, treatment and outcome.

Baseline routine blood examinations included complete blood count, high-sensitivity C-reactive protein, procalcitonin, lactate dehydrogenase (LDH), creatinine, aspartate aminotransferase, alanine transaminase, ferritin, prothrombin time, partial thromboplastin and D-dimer. Radiography and computerized tomography were used for chest imaging. On admission, each patient was scored for quick sequential organ failure assessment (qSOFA), Glasgow coma score and neutrophil-lymphocyte ratio. (*9*, *10*)

Standard of care was based on national guidelines that were continuously being updated during the study period. (*11*) Medications such as antiviral drugs and immunomodulators were not consistently available.

The severity of COVID-19 disease was categorized as asymptomatic, mild, moderate, severe and critical. Patients were labelled “asymptomatic” if they had no symptoms; “mild” if they had constitutional and nonspecific symptoms; “moderate” if they had pneumonia but did not require oxygen; “severe” if they had pneumonia plus hypoxemia, tachypnoea or hypotension; and “critical” if they had worsening pneumonia, sepsis or septic shock. (*11*)

In our analysis, we explored clinical characteristics and outcomes (frequency and type of complication, recovery rate and mortality) and identified factors associated with mortality in COVID-19 patients. Median, means, standard deviations and proportions were used to summarize the data. The *t*-test and χ^2^ test were used to test for differences in means and proportions, respectively. The Mann–Whitney U test was used to compare differences in median values. Fisher’s exact test or the χ^2^ test was used to examine differences between categorical data. A stepwise analysis model using multiple logistic regression was used to determine which variables were associated with mortality. Variables that were statistically significant (*P* < 0.05) in the univariate analysis were selected. Although both disease severity and qSOFA were statistically significant at the univariate level, only the former was included in the final model because these two variables had overlapping definitions. EPI-Info version 7.2.4.0 was used to process the data.

## Results

### Characteristic of cases at hospital admission

The mean age of the 280 COVID-19 patients was 48.4 years and the majority (64%) were females. Two thirds (63%) were aged under 60 years. More than half (62%) had exposure to a known case through either travel or close contact. The majority (58%, 161/280) of cases had at least one comorbidity, and 34% (94/280) had two or more comorbidities, with hypertension, cardiovascular disease (CVD) and diabetes being the most frequent. Pregnant patients comprised 16% of the cases and health care workers 23% (**Table 1A**). Among pregnant patients, 71% were in their third trimester of pregnancy.

**Table 1:** A. Demographic characteristics of adult COVID-19 patients admitted to Baguio General Hospital and Medical Center from 1 March to 27 October 2020

Clinical characteristics	Total, *n*(%)	Recovered, *n*(%)	Died, *n*(%)	*P*
Total number of patients	280	267	13	-
Age, years				
Mean ± SD	48.4 ± 18.5	47.7 ± 18.5	62.2 ± 13.5	0.71
18–44	131 (46.8)	129 (48.3)	2 (15.3)	*0.01*
45–59	44 (15.7)	43 (16.1)	1 (7.7)	-
60–79	98 (35.0)	88 (33.0)	10 (76.9)	-
^3^80	7 (2.5)	7 (2.6)	-	-
Sex				
Female	179 (64.0)	174 (65.2)	5 (38.5)	0.05
Male	101 (36.1)	93 (34.8)	8 (61.5)	-
Comorbidities	161 (57.5)	148 (55.4)	13 (100)	*< 0.01*
Hypertension	124 (44.3)	114 (42.7)	10 (76.9)	0.02
Diabetes mellitus	47 (17.0)	45 (16.9)	2 (15.4)	0.62
Cardiovascular disease	34 (12.1)	26 (9.7)	8 (61.5)	*< 0.01*
Bronchial asthma	17 (6.1)	16 (6.0)	1 (7.7)	0.57
Malignancy	12 (4.3)	12 (4.5)	-	-
Chronic kidney disease	4 (1.4)	1 (0.4)	3 (23.1)	*< 0.01*
Chronic obstructive pulmonary disease	3 (1.1)	3 (1.1)	-	-
Number of comorbidities				
0	119 (42.5)	119 (44.6)	-	*< 0.01*
1	68 (24.3)	65 (24.3)	3 (23.1)	-
2	66 (23.6)	59 (22.1)	7 (53.9)	-
> 2	27 (9.6)	24 (9.0)	3 (23.1)	-
Patient reported symptoms	164 (58.6)	153 (57.3)	11 (84.6)	0.04
Cough	111 (39.6)	101 (37.8)	10 (76.9)	*< 0.01*
Cold	49 (17.5)	48 (18.0)	1 (7.7)	0.30
Fever	40 (14.3)	35 (13.1)	5 (38.5)	0.03
Malaise	37 (13.2)	31 (11.6)	6 (46.2)	*< 0.01*
Dyspnoea	35 (12.5)	28 (10.5)	7 (53.9)	0.27
Sore throat	26 (9.3)	26 (9.7)	-	-
Headache	24 (8.6)	24 (9.0)	-	-
Anosmia	17 (6.1)	17 (6.4)	-	-
Dysgeusia	14 (5.0)	14 (5.2)	-	-
Anorexia	12 (4.3)	10 (3.8)	2 (15.4)	0.10
Diarrhoea	11 (3.9)	11 (4.1)	-	-
Chills	4 (1.4)	2 (0.8)	2 (15.4)	*0.01*
Seizure	2 (0.7)	2 (0.8)	-	-
Disease severity at admission based on national COVID-19 case definitions				
Asymptomatic	43 (15.4)	43 (16.1)	-	-
Mild	77 (27.5)	76 (28.5)	1 (7.1)	*< 0.01*
Moderate	126 (45.0)	123 (46.1)	3 (23.1)	-
Severe	23 (8.2)	21 (7.9)	2 (15.4)	-
Critical	11 (3.9)	4 (1.5)	7 (53.8)	-
Quick sequential organ failure assessment (qSOFA) score				
0	228 (81.4)	225 (84.3)	3 (23.1)	*< 0.01*
1	46 (16.4)	39 (14.6)	7 (53.9)	-
2	5 (1.8)	3 (1.1)	2 (15.4)	-
3	1 (0.4)	0 (0.0)	1 (7.7)	-
Glasgow coma score < 15	4 (1.4)	1 (0.4)	3 (23.1)	*< 0.01*
Respiratory rate (*3*)22 breaths/min	32 (11.4)	24 (9.0)	8 (61.5)	*< 0.01*
Systolic blood pressure £100 mmHg	23 (8.2)	20 (7.5)	3 (23.1)	0.08

*P*-values < 0.05 are italicized.

Upon admission, 59% of patients reported symptoms, most commonly respiratory (cough, cold or dyspnoea) and constitutional (fever or malaise) in nature. The other 41% did not report symptoms on admission. Twenty-one per cent of patients were observed to have tachypnoea, hypotension or altered mental state. Six patients (2.2%) had a qSOFA score of at least 2 (**Table 1A**).

Forty-five per cent of patients were assessed against the national case definitions as having moderate disease. Concomitant non-pulmonary syndromes such as stroke and myocardial infarction were noted (**Table 1A**).

Most patients (93.6%) had procalcitonin < 0.5 ng/mL. Many had high-sensitivity C-reactive protein > 10 ng/mL (37%) and ferritin > 341 ng/mL (42%). A few had elevations in other inflammatory markers such as LDH, aspartate aminotransferase, alanine transaminase and D-dimer, whereas anaemia, leukopenia and thrombocytopenia were not typical (**Table 1B**).

**Table 1:** B. Pertinent baseline diagnostic test results of adult COVID-19 patients admitted to Baguio General Hospital and Medical Center from 1 March to 27 October 2020

Diagnostic test	Reference range	Total *n* (range/%)	Recovered *n* (range/%)	Died *n* (range/%)	*P*
**Serum**					
Haemoglobin (g/L) (*n* = 280)	120–160	141 (131–152)	141 (131–152)	140 (124–142)	0.56
< 120	-	21 (7.5)	20 (7.5)	1 (7.7)	0.65
Haematocrit (L/L) (*n* = 280)	0.37–0.47	0.4 (0.4–0.5)	0.4 (0.4–0.5)	0.4 (0.38–0.41)	0.37
^3^0.47		46 (16.4)	45 (16.9)	1 (7.7)	0.34
Leukocytes (10^9^/L) (*n* = 280)	5–10	7.5 (5.8–9.8)	7.5 (5.8–9.7)	8.0 (6.3–10.9)	0.47
< 4	-	14 (5.0)	14 (5.2)	–	-
Neutrophil–lymphocyte ratio	1–3	2.5 (1.6–22.8)	2.4 (1.6–3.5)	4.4 (3.2–8.6)	*< 0.01*
£3	-	185 (66.1)	182 (68.2)	3 (23.1)	*< 0.01*
> 3 to < 9	-	83 (29.6)	76 (28.5)	7 (53.9)	0.05
^3^9	-	12 (4.3)	9 (3.4)	3 (23.1)	*0.01*
Platelets (*n* = 279)	150–400	253.0 (198–313)	257.0 (202–316)	196.0 (158.5–211.5)	*< 0.01*
< 125	-	5 (1.8)	4 (1.5)	1 (8.3)	0.20
High-sensitivity C-reactive protein (mg/L) (*n* = 264)	< 5	5.0 (1.5–18.7)	4.7 (1.5–16.0)	83.6 (33.4–131.5)	*< 0.01*
5–10	-	33 (12.5)	33 (13.1)	–	-
> 10	-	98 (37.1)	87 (34.5)	11 (91.7)	*< 0.01*
Procalcitonin (ng/mL) (*n* = 236)	-	0.05 (0.02–0.12)	0.05 (0.02–0.11)	1.17 (0.13–1.81)	*< 0.01*
< 0.5	-	221 (93.6)	217 (96.4)	4 (36.4)	*< 0.01*
Lactate dehydrogenase (U/L) (*n* = 263)	< 247	216.3 (174.6–285.8)	214.9 (174.3–278.9)	407.6 (236.5–657.3)	*< 0.01*
> 400	-	19 (7.2)	12 (4.8)	7 (53.9)	*< 0.01*
Creatinine (mg/dL) (*n* = 278)	0.55–1.02	0.71 (0.60–0.86)	0.71 (0.60–0.85)	0.76 (0.71–2.6)	*0.04*
> 1.02	-	36 (13.0)	30 (11.3)	6 (46.2)	*< 0.01*
Aspartate aminotransferase (U/L) (*n* = 277)	< 35	29.3 (23.2–40.0)	28.8 (22.9–39.0)	52.1 (33.7–86.0)	*< 0.01*
> 95	-	12 (4.3)	9 (3.4)	3 (23.1)	*0.01*
Alanine transaminase (U/L) (*n* = 278)	< 35	29.6 (17.8–46.0)	28.8 (17.4–44.1)	42.9 (25.0–49.4)	0.09
> 95	-	17 (6.1)	15 (5.7)	2 (15.4)	0.18
Ferritin (ng/mL) (*n* = 190)	4–341	295.0 (68.1–653.7)	281.1 (63.5–604.8)	982.1 (238.7–1611.0)	*0.04*
> 341	-	80 (42.1)	75 (41.0)	5 (71.4)	0.11
Prothrombin time (seconds) (*n* = 266)	-	12.1 (11.5–12.8)	12.1 (11.4–12.7)	12.8 (12.3–18.7)	*< 0.01*
> 15.3	-	7 (2.6)	2 (0.8)	5 (38.5)	*< 0.01*
Partial thromboplastin time (seconds) (*n* = 263)	-	29.6 (27.7–31.8)	29.5 (27.7–31.8)	33.2 (27.1–39.7)	0.12
> 35	-	24 (9.1)	20 (8.0)	4 (33.3)	*0.02*
D-dimer (µg/mL) (*n* = 260)	< 0.5	0.54 (0.18–1.19)	0.52 (0.34–1.17)	0.94 (0.63–5.36)	*0.03*
> 1	-	61 (29.6)	57 (28.8)	4 (50.0)	0.18
**Imaging**					
Chest radiograph	-	*n* = 276	*n* = 263	*n* = 13	-
Patients with findings	-	151 (54.7)	140 (53.2)	11 (84.6)	*0.04*
Infiltrates	-	147 (97.4)	139 (99.3)	8 (72.7)	*< 0.01*
Effusion	-	2 (1.3)	1 (0.7)	1 (9.1)	0.11
Consolidation	-	3 (2.0)	1 (0.7)	2 (18.2)	*0.01*
Computed tomography	-	*n* = 174	*n* = 166	*n* = 8	-
Patients with findings	-	124 (71.3)	118 (71.1)	6 (75.0)	1.00
Ground glass opacity	-	111 (89.5)	105 (89.0)	6 (100)	1.00

***P*** values < 0.05 are italicized.

SD: standard deviation.

More than half of the population had chest radiography findings, with infiltrates being the most common. Computed tomography was available to two thirds (62%) of patients. Findings were noted in 71%, ground glass opacity being the most common (**Table 1B**).

### Illness outcomes

**Figure 1:**
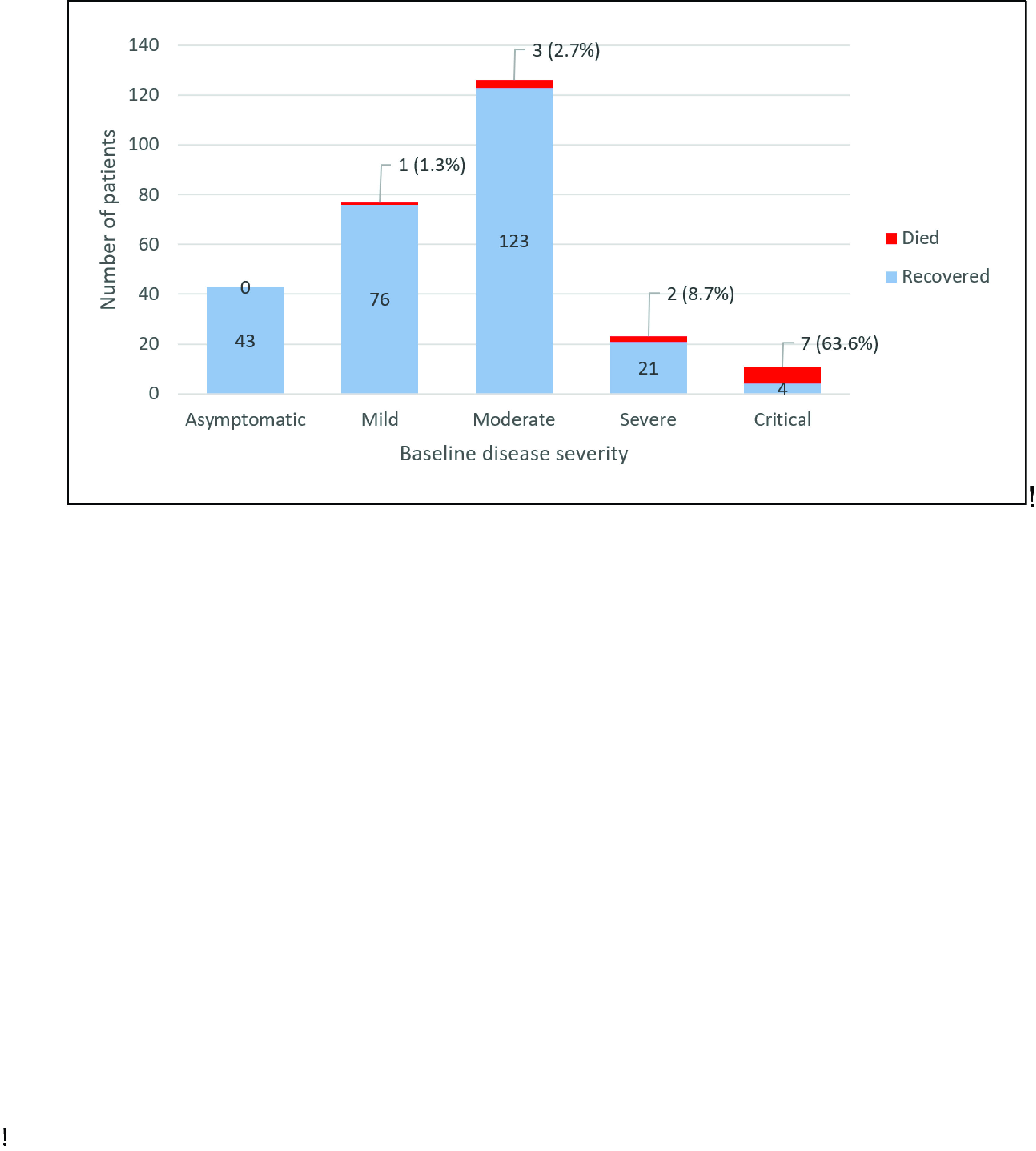
Outcomes of adult patients with COVID-19 based on disease severity on admission to Baguio General Hospital and Medical Center (n = 280)

The overall recovery rate was 95% (267/280), with most recovered cases having asymptomatic to moderate disease on admission. All health care workers and pregnant patients recovered. Mortality occurred in 5% (13/280) of patients, with the most common cause of death being multiorgan failure (39%, 5/13). Among those who died, most were males in the 60–79-year age group with at least one comorbidity, respiratory symptoms on admission, a qSOFA score (*3*)1 and bilateral lung involvement. Nine were assessed as having severe to critical disease at admission (**Fig. 1**).

[**insert** Figure 1]

**Figure 2:**
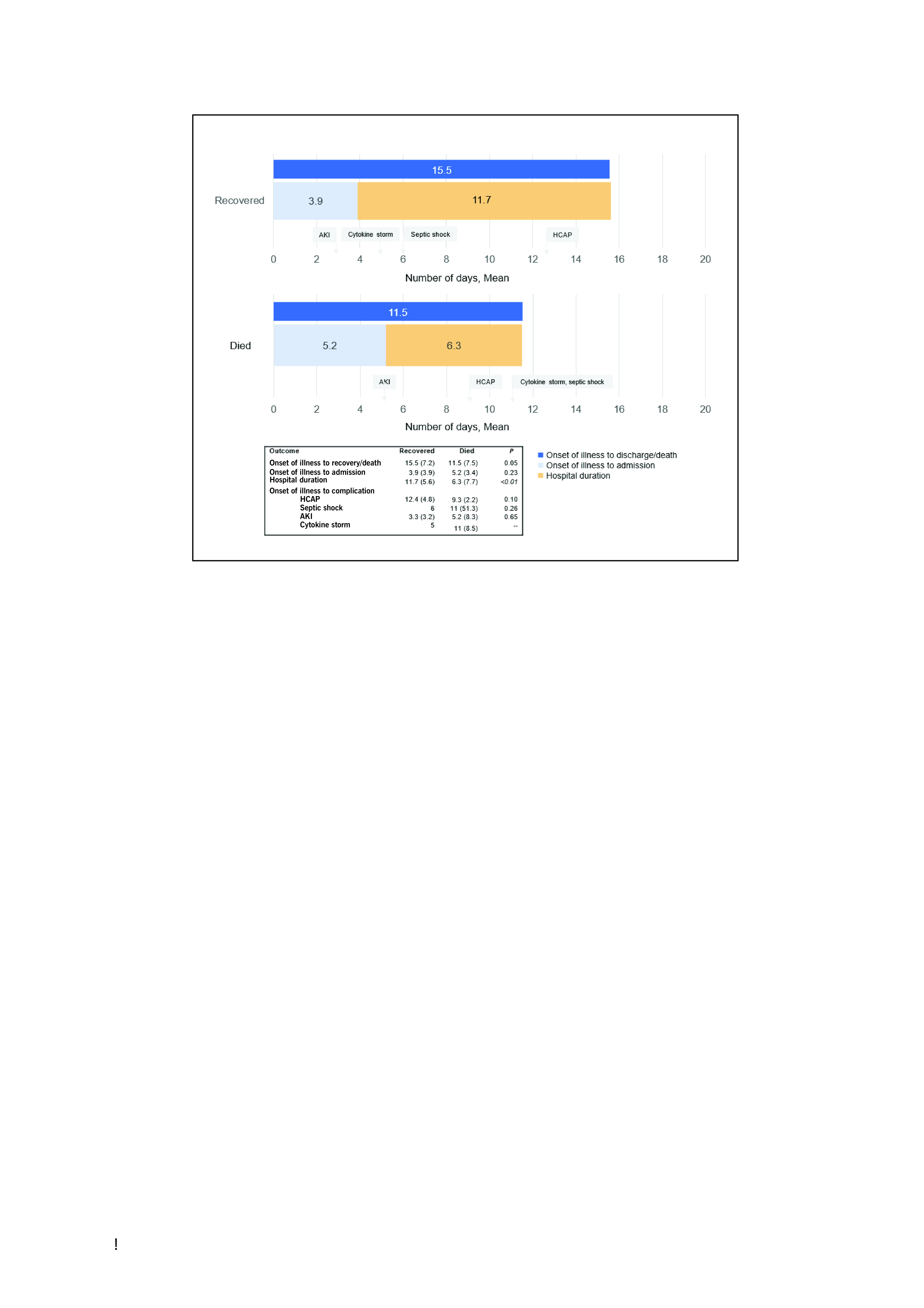
Mean duration (in days) of illness to admission, hospital duration, and onset of complications among patients admitted to Baguio General Hospital and Medical Center from 1 March to 27 October 2020

The mean time from illness onset to discharge from hospital for recovered patients was 15.5 days (range: 4.0–54.0) with the mean hospital stay being 11.7 (± 5.6) days (range: 3.0–49.0). For cases who died, the mean time from illness to death was 11.5 days (range: 4.0–29.0) (**Fig. 2**).

Forty-two (15%) cases had complications, most of whom had moderate to critical disease on admission (32/42) (**Table 2**, **Fig. 2**). Health care-associated pneumonia was the most frequent complication. Among the 14 patients who developed acute kidney injury, six underwent haemodialysis and none of those six survived. Among patients with complications, 30 (71%) recovered and 12 (29%) died. Among those who died, many had cardiovascular or renal complications or secondary infections (**Table 2**).

[**insert** Figure 2]

**Table 2:** Frequency of complications in adult COVID-19 patients admitted to Baguio General Hospital and Medical Center from 1 March to 27 October 2020

Complications	Total *n*(%)	Recovered *n*(%)	Died *n*(%)	*P*
Total number of patients	280	267	13	-
Number of patients with complications	42 (15.0)	30 (11.2)	12 (92.3)	*< 0.01*
Secondary infection	22 (7.9)	16 (6.0)	6 (46.2)	*< 0.01*
HCAP	17 (6.1)	13 (4.9)	4 (30.8)	*< 0.01*
Septic shock	6 (2.1)	2 (0.8)	4 (30.8)	*< 0.01*
Bacteraemia	3 (1.1)	3 (1.1)	-	-
CAUTI	1 (0.4)	-	1 (7.7)	-
Acute kidney injury	14 (5.0)	5 (1.9)	9 (69.2)	*< 0.01*
Cardiovascular	11 (3.9)	2 (0.8)	9 (69.2)	*< 0.01*
Myocardial infarction	7 (2.5)	1 (0.4)	6 (46.2)	*< 0.01*
Fatal arrhythmia	7 (2.5)	1 (0.4)	6 (45.2)	*< 0.01*
Transaminitis	11 (3.9)	10 (3.8)	1 (7.7)	0.41
Haematologic/immunologic	8 (2.9)	4 (1.5)	4 (30.8)	*< 0.01*
Cytokine storm	4 (1.4)	2 (0.8)	2 (15.4)	*0.01*
Thrombocytopenia	3 (1.1)	1 (0.4)	2 (15.4)	*0.01*
Leukopenia	1 (0.4)	1 (0.4)	-	-
Neurological	2 (0.7)	-	2 (15.4)	-
Seizure	1 (0.4)	-	1 (7.7)	-
Stroke (ischaemic)	2 (0.7)	-	2 (15.4)	-

***P*** values < 0.05 are italicized.

CAUTI: catheter-associated urinary tract infection; HCAP: health care-associated pneumonia.

### Treatment of cases

Antibiotics were prescribed for 73% of cases and antiviral drugs for 55% of cases (**Table 3**). The most common antiviral drugs used were oseltamivir (83/154), favipiravir (54/154), remdesivir (16/154) and lopinavir-ritonavir (1/154). Hydroxychloroquine was administered during March–May 2020, while steroids, particularly dexamethasone, were prescribed to patients from August 2020. Supplemental oxygen was used in 11% of cases (**Table 3**). Among the seven cases who underwent renal replacement therapy, only one had underlying chronic kidney disease. Patients with extrapulmonary syndrome such as stroke, myocardial infarction and seizure were treated according to guidelines for the general population.

**Table 3:** Treatment modalities of adult COVID-19 patients admitted to Baguio General Hospital and Medical Center from 1 March to 27 October 2020

Treatment	Total *n*(%)	Recovered *n*(%)	Died *n*(%)	*P*
Total number of patients	280	267	13	-
Antibiotics	203 (72.5)	192 (71.9)	11 (84.6)	0.26
Antivirals	154 (55.0)	149 (55.8)	5 (38.5)	0.17
Immunomodulators	70 (25.0)	61 (22.9)	9 (69.2)	*< 0.01*
Hydroxychloroquine	25 (8.9)	24 (9.0)	1 (7.7)	0.67
Corticosteroids	45 (16.1)	37 (13.9)	8 (61.5)	*< 0.01*
Intravenous immunoglobulin	4 (1.4)	3 (1.1)	1 (7.7)	0.17
Tocilizumab	3 (1.1)	2 (0.8)	1 (7.7)	0.13
Oxygen support	32 (11.4)	24 (9.0)	8 (2.9)	*< 0.01*
Nasal cannula	24 (8.6)	21 (7.9)	3 (23.1)	0.09
Face mask	3 (1.1)	1 (0.4)	2 (15.4)	*0.01*
Invasive mechanical ventilation	5 (1.8)	2 (0.8)	3 (23.1)	*< 0.01*
Renal replacement therapy	7 (2.5)	1 (0.4)	6 (46.2)	*< 0.01*
Haemodialysis	5 (1.8)	1 (0.4)	4 (30.8)	*< 0.01*
Haemodialysis with haemoperfusion	2 (0.7)	-	2 (15.4)	-

***P*** values < 0.05 are italicized.

### Mortality from COVID-19

Using multiple logistic regression with a stepwise analysis model, factors associated with mortality in patients with COVID-19 were chronic kidney disease, CVD, prothrombin time > 15.3 seconds and LDH > 400 (**Table 4**).

**Table 4:** Factors associated with mortality of adult COVID-19 patients admitted to Baguio General Hospital and Medical Center from 1 March to 27 October 2020

Variables	Adjusted odds ratios	95% confidence interval	*P*-value
Presence of chronic kidney disease	324.7	12.5 to 8 456.4	*0.001*
Presence of cardiovascular disease	10.6	1.7 to 66.8	*0.012*
Prothrombin time (*3*)15.3 second	74.6	3.6 to 1 562.6	*0.006*
Lactate dehydrogenase > 400	26.4	3.8 to 184.6	*0.001*

***P*** values < 0.05 are italicized.

## Discussion

Our study assessed the clinical profile and outcomes of hospitalized adult COVID-19 patients in a single hospital in Baguio City, Philippines. The COVID-19 cases comprised mostly female patients with a mean age of 48.4 years. Moderate, severe and critical disease made up 45%, 8% and 4% of the COVID-19 patients, respectively. The recovery rate was 95% and mortality was associated with having chronic kidney disease, CVD, elevated LDH and prolonged prothrombin time at hospital admission.

The female-to-male ratio in our study was 1.8:1, yet 62% of cases that died were male. Several other studies have shown a male predominance of COVID-19 cases, (*12*, *13*) and a recent meta-analysis showed that male sex was significantly associated with severe disease. (*14*) However, in our study, there was no significant difference in sex between the cases that recovered and those that died. The high female-to-male ratio in our study may have been due to the former outnumbering the latter in all age groups except for those aged 1–4 years in Baguio City. (*4*)

In our study, 77% of COVID-19 cases that died were aged 60–79 years, reflecting national data, whereby 60% of confirmed deaths were males aged at least 60 years. (*15*) Old age is a known risk factor for severe COVID-19, for reasons not yet fully understood. (*16*, *17*) Changes in the immune system and prevalence of comorbidities in this age group contribute to the risk.

WHO recognizes that underlying comorbidities can negatively impact outcomes in COVID-19 patients, (*18*) with confirmed COVID-19 patients with comorbidities having increased admission rates to intensive care units and mortality. (*19*) Although all the cases in our study who died had at least one comorbidity, the presence of a comorbidity did not in itself significantly increase the likelihood of death. However, having chronic kidney disease and CVD were significantly associated with mortality. Chronic kidney disease is considered the most prevalent risk factor for severe COVID-19 worldwide, especially for patients with an estimated glomerular filtration rate < 30 mL/min/1.73 m^2^. (*17*, *20*) In addition to chronic kidney disease, a higher proportion of those who died also had acute renal complications warranting haemodialysis. It is hypothesized that kidney involvement is through direct cellular and immune-mediated damage due to the presence of the virus. (*21*) COVID-19 patients presenting with acute kidney injury have been shown to have a higher risk of death than patients with acute kidney injury from other conditions. (*22*) A recent meta-analysis found that pre-existing CVD is also an independent risk factor associated with poor outcomes from COVID-19. (*23*) Patients who have pre-existing comorbidities or present with complications should be closely monitored for severe outcomes. This, in combination with evidence relating to other complications during COVID-19 infection (e.g. hospital-acquired infections), supports the rapidly accumulating evidence that COVID-19 may have multisystemic affectations.

Our study found an association between mortality and prolonged prothrombin time (> 15.3 seconds) and elevated LDH (> 400). Several studies have shown that a prolonged prothrombin time is associated with a poorer outcome among COVID-19 patients. (*24*, *25*) Coagulation parameters not only reflect haemostasis but are also associated with the inflammation and organ dysfunction brought about by COVID-19 infection. In a pooled analysis, elevated LDH values were associated with a 6-fold increase in odds of severe COVID-19 disease and > 16-fold increase in odds of mortality. (*26*) Since LDH is present in lung tissue, patients with severe COVID-19 infections who present with a severe form of interstitial pneumonia can be expected to release greater amounts of LDH in the circulation.

High baseline levels of inflammatory biomarkers (e.g. serum LDH, alanine transaminase and D-dimer) are considered poor prognostic factors that are associated with mortality, increased stay in the intensive care unit and severe disease. (*11*) Certain haematological abnormalities (e.g. decreased haemoglobin, white blood cell count and platelets), although not rare in COVID-19, are seen in severe disease. (*27*) Both scenarios were seen in a minority of our cases. This may relate to our population’s low mortality rate. Meanwhile, a low or normal procalcitonin level, observed in a high number of patients in our study, is compatible with a viral infection. Elevated levels may be due to other non-viral, even non-infectious, causes. (*11*)

That 73% of our patients received antibiotics is a concern, although this was mainly as a preventive measure and due to many patients having a secondary infection, including hospital-acquired pneumonia, bacteraemia and complicated urinary tract infections. Secondary infections can contribute to a poorer outcome, and when faced with severely ill hospitalized patients where the diagnosis of a bacterial superinfection is uncertain, antibiotics are often started. (*28*) Because this study was in the early phase of the pandemic, hydroxychloroquine and lopinavir-ritonavir were included among the investigational drugs given to patients.

The most common symptoms in our COVID-19 patients were cough, cold, fever, dyspnoea and malaise. Although, in the univariate analysis, the proportions reporting cough, fever and malaise were significantly higher in cases that died than in those that recovered, these proportions were not associated with mortality in multivariate analysis. Other studies have identified various symptoms as prognosticators for mortality. Dyspnoea was consistently identified as a risk factor for mortality in multinational meta-analyses involving thousands of patients. (*29*, *30*) In contrast, a meta-analysis involving > 50 000 patients in 13 countries showed that headache, diarrhoea, vomiting and cough indicate a lower risk of death. (*29*) In addition, anosmia and dysgeusia are peripheral neurological symptoms of COVID-19 that have been investigated for their association with recovery, with studies on anosmia reporting it as being inversely associated with hospitalization and as a marker of milder COVID-19 disease. (*31*, *32*) Conversely, a meta-analysis showed that olfactory and taste dysfunction had no bearing on severity of COVID-19 disease. (*33*) In our study, all patients presenting with dysgeusia and anosmia recovered. Differences in study definitions, study methodologies and tools for detecting anosmia and dysgeusia may account for the differences in results.

Pregnancy is now recognized as a risk factor for contracting COVID-19. A weakened immune system during pregnancy confers a higher risk of infection with SARS-CoV-2. (*34*) In this study, 45 patients were pregnant but none died. Possible causes for this low mortality rate could be the lower age of pregnant patients as well as the lower rate of concomitant comorbidity in this subgroup.

Our study had some limitations. First, the study design was cross-sectional; causal inference and associations may be inherently difficult to make and interpret because the outcome, exposure and investigated risk factors were collected simultaneously. The frequency and type of complications seen in our patients cannot be wholly attributed to the effects of COVID-19. Second, the selection of our study population was non-randomized, and data analysis was non-stratified and non-matching. Although multiple logistic regression was used to identify risk factors associated with mortality, our sample size was small, leading to wide confidence intervals. Therefore, caution should be applied when interpreting the results. At the time of writing, the pandemic is ongoing and the clinical profile and prognosis of COVID-19 patients in our institution may change over time.

In conclusion, most of the patients in our population were classified with asymptomatic to moderate disease on admission and few had complications. Overall, 95% of cases recovered and 5% died. The presence of chronic kidney disease, CVD, elevated LDH and prolonged prothrombin time were associated with mortality in our population. Based on these results, we strongly recommend that patients with comorbidities, including pregnancy and those of older age, should take all necessary precautions to avoid getting infected with SARS-CoV-2.
